# Moroccan Medicinal Plants Used to Treat Cancer: Ethnomedicinal Study and Insights into Pharmacological Evidence

**DOI:** 10.1155/2022/1645265

**Published:** 2022-10-25

**Authors:** Naoufal El Hachlafi, Nesrine Benkhaira, Mohamed Ferioun, Fahd Kandsi, Mohamed Jeddi, Abderrahim Chebat, Mohamed Addi, Christophe Hano, Kawtar Fikri-Benbrahim

**Affiliations:** ^1^Laboratory of Microbial Biotechnology and Bioactive Molecules, Faculty of Sciences and Technologies Faculty, Sidi Mohamed Ben Abdellah University, P.O. Box 2202, Imouzzer Road, Fez, Morocco; ^2^Laboratory of Natural Resources and Environmental, Faculty of Polydisciplinary of Taza, Sidi Mohamed Ben Abdellah University, Fez, Morocco; ^3^Laboratory of Bioresources, Biotechnology, Ethnopharmacology and Health, Faculty of Sciences, Mohammed First University, B. P. 717, Oujda 60000, Morocco; ^4^Moroccan Anti Poison and Pharmacovigilance Center, P.O. Box 6671, Rabat, Morocco; ^5^Laboratoire d'Amélioration des Productions Agricoles, Biotechnologie et Environnement (LAPABE), Faculté des Sciences, Université Mohammed Premier, Oujda 60000, Morocco; ^6^Laboratoire de Biologie des Ligneux et des Grandes Cultures, INRAE USC1328, Uni-versity of Orleans, CEDEX 2, Orléans 45067, France; ^7^Le Studium Institute for Advanced Studies, 1 Rue Dupanloup, Orléans 45000, France

## Abstract

Cancer is one of the major medical challenges, with an unacceptably high death toll worldwide. In Morocco, medicinal plants continue to play a pivotal therapeutic role despite the development of modern sanitation systems. In the current study, an ethnobotanical survey was carried out at the Moroccan national institute of oncology, Rabat, and we aimed at (1) establishing an exhaustive inventory of indigenous knowledge of Moroccan medicinal plants used to manage cancer and (2) confirming the reported ethnopharmacological uses through bibliometric review. An ethnobotanical survey was conducted with 291 cancer patients at the Moroccan National Institute of Oncology, Rabat, during a period of 4 months, from February to May 2019, through semistructured interviews. Ethnobotanical indices, including informant consensus factor (FIC), use report (UR), relative frequency citation (RFC), botanical family use value (FUV), fidelity level (FL), and index of agreement on remedies (IAR), were employed in data analyses. The survey revealed that 39 medicinal plants belonging to 27 botanical families and 38 genera were used to treat cancer. The most used ethnospecies were *Aristolochia longa* with the highest RFC value (0.096), followed by *Nigella sativa*, *Ephedra alata*, *Euphorbia resinifera*, and *Lavandula dentata*, éwith RFC values of 0.072, 0.054, 0.044, and 0.044, respectively. In regard to the plant families, Lamiaceae contributed the highest number of plants with five species (FUV = 0.034), followed by Asteraceae (4 species; FUV = 0.020), and Fabaceae (4 species; FUV = 0.020). The leaves are the most popular plant part used by the studied population against cancer; otherwise, decoction was the most commonly used method for remedy preparation and the highest FIC was noticed for uterine cancer treatment (0.86). Considering these findings, further investigations into the recorded plant species should be performed to assess phytochemical constituents and pharmaceutical benefits in order to identify their active compounds for any drug formulations.

## 1. Introduction

Cancer is one of the leading causes of death in humans [1, 2]. According to the World Health Organization (WHO) and the International Agency for Research on Cancer (IARC), there were approximately 9.6 million deaths and 18.1 million new cancer cases worldwide in 2018 [3]. The estimated number of new cancer cases and deaths worldwide by 2040 is around 27.5 million and 16.3 million, respectively [4]. In Morocco in 2020, there were 59,370 new cases and 35,265 cancer deaths for both sexes and all ages. Breast cancer was the most common type of cancer in Morocco (19.8%), followed by lung (12.4%), colorectal (7.7%), prostate (7.5%), non-Hodgkin's lymphoma (4.1%), and other cancers (48.5%) [5].

To date, chemotherapy, surgery, and radiotherapy remain the most frequently employed options for cancer treatment, but their toxicity and side effects restrict their usage [6, 7]. Chemotherapy and/or radiotherapy may lead to some serious complications, such as fatigue, chronic pain, oral mucositis, anorexia, gastrointestinal toxicity, hepatotoxicity, nephrotoxicity, insomnia, edema, depression/anxiety, or constipation [6, 7]. These effects are difficult to manage and can significantly affect the quality of a patient's life [2].

Furthermore, due to poverty and the high cost of biological analysis and treatment, access to this type of treatment remains limited in rural Morocco and other developing countries [6]. As a result, cancer has become one of the medical challenges that has resulted in an unacceptably high death toll in these areas [8, 9]. Thus, there is a need to revert to homegrown solutions, such as exploring the flora of our own country, Morocco, which is part of the North African botanical block and is home to approximately 4,200 taxa, 32% (1350 taxa) of which are endemic [9].

In practice, 55% to 90% of the general population frequently reports using medicinal plants to treat Moroccan cancer patients [2]. Several ethnobotanical studies conducted in Morocco have also revealed significant traditional uses of medicinal plants for cancer management. Aboufaras et al. recently conducted a survey with 530 cancer patients at Beni Mellal's Regional Oncology Center. They reported that *Aristolochia longa* L., *Euphorbia resinifera* Berg, and *Nigella sativa* L. are recommended for the treatment of a variety of cancers, including breast, cervix, lung, colorectal, oral, stomach, and bladder cancers [2].

Furthermore, many plant-derived anticancer agents, such as vinca alkaloids (vinblastine, vincristine, and vindesine), epipodophyllotoxins (etoposide and teniposide), taxanes (paclitaxel and docetaxel), and camptothecin derivatives (camptothecin and irinotecan), have been used successfully in clinical studies [10–13].

As a result, it is critical to identify natural agents present in medicinal plants that may have the anticarcinogenic potential [1, 14–18]. Because ethnobotanical and ethnopharmacological surveys, including those conducted with patient interviews, are effective methods for documenting and identifying medicinal plants used in traditional pharmacopeia systems, the current study was carried out at the Moroccan National Institute of Oncology (Sidi Mohamed Ben Abdellah) to record medicinal plant species used in the management of various types of cancer and to identify the potential bioactive phytochemicals in the claimed plants as well as their potential mechanisms of action via an in-depth literature revision.

## 2. Materials and Methods

### 2.1. Study Area

**Figure 1:**
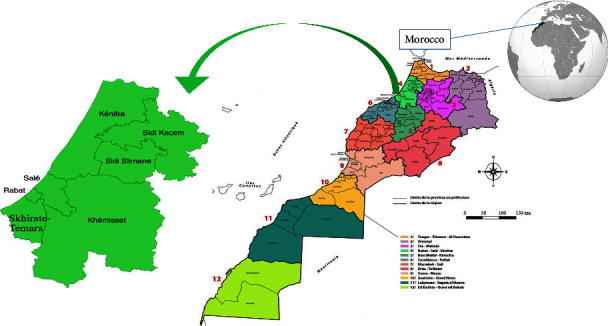
Geographical location of the study area.

Morocco's Rabat-Sale-Kenitra region is in the northwest (34° 02′ 00″ N, 6° 50′ 00″ W) (Figure 1). It has a population of about 4,580,866 people and an area of 18,194 km^2^ (2.56% of the national territory). The Tangier-Tetouan-Al-Hoceima region borders it on the north, the Fez-Meknes region on the east, the Beni Mellal-Khenifra and Casablanca-Settat regions on the south, and the Atlantic Ocean on the west [19].

The study area has three prefectures: Rabat, Sale, and Skhirate-Temara, and it has four provinces: Kenitra, Sidi Slimane, Khemisset, and Sidi Kacem. The Rabat-Sale-Kenitra region concentrates the majority of the Kingdom's demographic, economic, administrative, and cultural flows. This development is mainly due to the administrative weight of Rabat, the capital of Morocco. Its exceptional geographical position, its human resources, and its natural potential make it a platform of development and a crossroad for investment and partnership [19].

The Rabat-Sale-Kenitra region has a remarkable natural and environmental heritage with numerous sites playing a major role in the conservation of biodiversity and ecosystems. This region contains over 408 vascular plant species and subspecies from 261 genera and 62 botanical families, accounting for approximately 10% of the Moroccan vascular flora [20, 21]. Remarkably, this region contains many natural reservoirs, such as the Mamora forest, which is one of the world's most important ecological reserves of *Quercus suber* [16].

### 2.2. Ethnobotanical Fieldwork

In order to collect and document indigenous knowledge of Moroccan medicinal plants used to manage cancer, a series of ethnobotanical surveys were carried out at the Moroccan national institute of oncology, Rabat (Sidi Mohamed Ben Abdellah). The target population was 1216 cancer patients who consulted consecutively during the study period. The size of the sample was estimated using the following formula [2]:

(1) $$\begin{array}{c} n=\frac{z^{2}\times p\left(1-p\right)/e^{2}}{1+\left(z^{2}\times p\left(1-p\right)/e^{2}N\right)}\text{,} \end{array}$$

where *n*: Sample size; *N*: population size = 1216, *z*: *z*-score = 1.96; *e*: error margin = 5%; and *p*: standard deviation = 0.5.

The minimum sample size estimated was 291 participants, with a confidence level of 99%. Indeed, among 1216 (*N*) patients, 291 (*n*) informants with different types of cancer were selected randomly. No special selection criteria were used in the choice of the participants.

The data were collected during a period of 4 months, from February to May 2019, through semistructured interviews using the Moroccan Arabic dialect, and the time spent in each interview varied from 20 to 30 min. This survey was designed first to establish the sociodemographic features of cancer patients (name, age, gender, education level, ethnicity, place of residence, and income of participants) and the traditional remedies used in cancer treatment. In fact, the questionnaire included different ethnobotanical and ethnomedicinal information such as vernacular and scientific names; plant families; plant parts used; preparation methods; administration modes; posology; side effects; and therapeutic combinations.

### 2.3. Species Identification, Systematization, and Preservation

The species taxonomic identification was undertaken based on standard Moroccan floras, including the practice flora of Morocco [22], vascular flora of Morocco, inventory and chorology [23], traditional Moroccan pharmacopeia [24], and Moroccan medicinal plants [25]. The botanical names of inventoried species were checked at the Phytovigilance Department of Moroccan Poison Control and the Pharmacovigilance Center, Rabat, from online botanical databases, namely: The Plant List (https://www.theplantlist.org/), African Plant Database (https://www.ville-ge.ch/musinfo/bd/cjb/africa/recherche.php) and the International Plant Names Index (https://www.ipni.org).

The listed plant species with their correct nomenclature were arranged alphabetically by family names based on the Angiosperm Phylogeny Group-IV (APG-IV) classification 2016 and vernacular names. Moreover, an EPPO code for each plant species was provided from an online database, the European and Mediterranean Plant Protection Organization (https://www.eppo.int). An EPPO code is an encoded identifier of species names, both scientific and vernacular. Finally, all the preserved specimens in the form of dried plants, parts of plants or pressed plant samples have been deposited in the herbarium of the Department of Biology, Faculty of Sciences and Technologies, University of Sidi Mohamed Ben Abdellah Fez, with defined ID numbers (BLMUP).

### 2.4. Quantitative Data Analysis

To describe the collected ethnopharmacological information, data were first sorted in the Microsoft Excel database and arranged in use reports (UR, a citation of one plant used by one respondent). Then, the one-way analysis of variance (ANOVA) was used to analyze the socio-demographic data of the patients in order to determine whether there were any significant variations between the means as well as correlations between the variables (Independent Samples T-Test, *p* values ≤0.05 were considered statistically significant). Ethnobotanical quantitative indices were also used to discuss our findings and to clearly indicate their novelty value compared with other ethnobotanical studies, which included the informant consensus factor (FIC), the use report (UR), the relative frequency citation (RFC), the botanical family use value (FUV), the fidelity level (FL), and the index of agreement on remedies (IAR). Moreover, principal component analysis (PCA) was performed based on the ethnobotanical indices using XLSTAT statistics version 2016 software.

#### 2.4.1. Informant Consensus Factor (FIC)

The FIC shows the homogeneity of the exchange of traditional knowledge between informants concerning the use of taxa to treat various disease categories. FIC was calculated using the following formula [26]:

(2) $$\begin{array}{c} FIC=\frac{Nur-Nt}{Nur-1}\text{,} \end{array}$$

where Nur refers to the number of usage reports for each ailment (type of cancer) and Nt is the number of species used for the same ailment. A high value (close to 1) of FIC indicated that the reported plant is primarily recognized as an anticancer agent by the informants.

With (0 < FIC < 1).

#### 2.4.2. Fidelity Level (FL)

The fidelity level (FL) indicates the effective effects of a given plant species against a particular ailment. FL was calculated according to Sreekeesoon & Mahomoodally [27].

(3) $$\begin{array}{c} FL=\frac{Ip}{Lu}\times 100\text{,} \end{array}$$

where Ip is the number of informants who used a specific species for a particular type of cancer, and Lu is the total number of respondents who indicated all uses of the given species for the treatment of any cancer.

#### 2.4.3. Relative Frequency Citation (RFC)

The relative citation frequency (RFC) was calculated by dividing the citation frequency (FC) by the population size (*N*) [28].

(4) $$\begin{array}{c} RFC=\frac{FC}{N}\text{.} \end{array}$$

This index demonstrates the local relative importance of each species.

#### 2.4.4. Family Importance Value (FIV)

The family importance value (FIV) shows the significance of botanical families. It was established to evaluate the biological taxonomic value of species and was calculated as follows [27]:

(5) $$\begin{array}{c} FIV=\frac{RFC}{Ns}\text{,} \end{array}$$

where RFC = the use value of the species belonging to the family; Ns = the number of species in the family.

#### 2.4.5. Index of Agreement on Remedies (IAR)

The index of agreement on remedies (IAR) was designed to measure the value of species for which there is consensus on more than one traditional use. Indeed, a species with a high number of citations in several cancers may rank higher than plants with more citations in any single type of cancer. This index was calculated according to the following formula [29]:

(6) $$\begin{array}{c} IAR=\frac{Nr-Na}{Nr-1}\text{.} \end{array}$$

With Nr, the total number of use citations for a specific plant across all use categories, and Na, the number of mentioned categories.

### 2.5. Ethical Statement and Consent to Participate

This study has been carried out with the permission of the committee for ethical research of the Moroccan Poison Control and Pharmacovigilance Center and the Moroccan National Institute of Oncology, under the number 310/2019. The data was collected with respect to confidentiality, anonymity, and consent. All patients were informed about the objective of this investigation. Oral and signed consent was obtained from the study participants.

### 2.6. Literature-Based Validation of the Data

Based on previous ethnobotanical investigations of Moroccan anticancer flora, we performed a bibliometric survey using different scientific search engines, including Google Scholar, Web of Science, Scopus, ScienceDirect, SpringerLink, Medline, SciFinder, and PubMed. The inventoried plants in this study were compared with other ethnobotanical surveys of plants used for cancer management in Morocco and other neighboring countries (Algeria, Tunisia, and Libya). This comparison allowed us to assess the originality and uniqueness of traditional uses sustained by the studied population and to report new plant citations and new medicinal practices against cancer. Furthermore, the data on the *in vitro* and *in vivo* anticancer properties of these plants, as well as the mechanisms underlying their activities, were summarized. Finally, the major chemical compounds demonstrating anticancer properties were also reviewed in this study, and ChemDraw Ultra 16.0 software was used to draw their chemical structures. The IUPAC names of the reported compounds were checked from PubChem databases (pubchem.ncbi.nlm.nih.gov).

## 3. Results and Discussion

### 3.1. Sociodemographic Data

**Figure 2:**
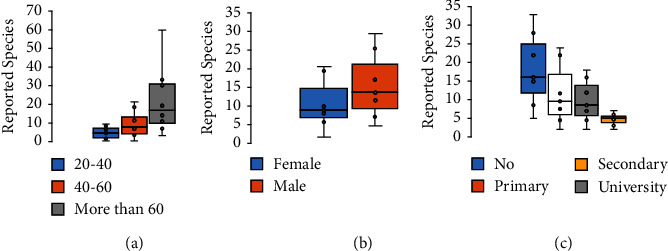
Number of reported ethnospecies variations according to (a) age; (b) gender; and (c) education level.

According to the age distribution (Table 1, Figure 2), 55% of respondents were between the ages of 40 and 60, followed by those between the ages of 20 and 40 (29%), and only a few informants were older than 60 years old (16%). According to our findings, people over the age of 40 were the most likely to use medicinal plants to treat various types of cancer. As a result, the difference in age and indigenous knowledge was statistically significant (*P*=0.001). These findings could be explained by the fact that older people have more knowledge about the use of medicinal plants and their benefits as a result of practical knowledge passed down from older generations [16, 30]. Other ethnobotanical surveys carried out on a national and international scale showed similar results [16, 30–32].

Among the 292 patients interviewed, 54% were women and 44% were men (Table 1, Figure 2). This demonstrates that women are more familiar with traditional medicine than men because women typically care for their families using medicinal plants as herbal remedies. These findings are consistent with those of other studies conducted in Messina, Morocco [33], Moulay Yacoub, Northeast Morocco [34], and Kinmen, Taiwan [31].

Our survey showed that most of the informants were illiterate (36%), 28% had a secondary level, 23% had a primary school education, and only 13% had a university level. These findings show that informants with lower levels of education have more frequent contact with medicinal plants, whereas those with higher levels of education have less exposure to medicinal plants and less relevant knowledge of traditional medicine. Several studies have found that illiterate people are more interested in medicinal plants than people with higher educational levels [2, 33–35].

In terms of habitat, findings revealed that 60% of informants lived in rural areas, while 40% lived in urban areas. People living in rural areas are closely associated with nature because of poverty and lack of access to health facilities. These findings are consistent with those reported in previous ethnobotanical surveys [36, 37].

This study showed that 51% of informants were in an intermediate socioeconomic situation, 44% were in a low socioeconomic situation, and only 5% were in a high socioeconomic situation. These findings are consistent with those of Benkhaira et al. [30]. This could be explained by the high cost of modern medical treatments, which creates a barrier to access and drives people to seek traditional treatments [38].

### 3.2. Diversity of Medicinal Plants Used to Treat Cancer

**Figure 3:**
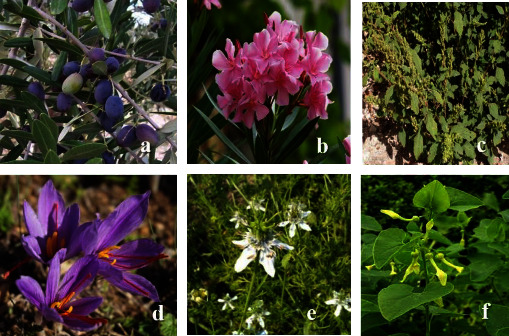
Main ethnospecies used against cancer by the informants. (a) *Olea europaea*, (b) *Nerium oleander*, (c) *Chenopodium ambrosioides*, (d) *Crocus sativus*, (e) *Nigella sativa*, and (f) *Aristolochia longa.*

In the present ethnopharmacological survey, 39 medicinal plants belonging to 27 botanical families and 38 genera were used to treat cancer. Ethnobotanical knowledge of the inventoried plants is mentioned in Table 2, which includes the data on botanical families, vernacular and scientific names, parts used, methods of preparation, traditional dosage, route of administration, and ethnobotanical indices. The most used ethnospecies were *Aristolochia longa L.* with the highest RFC value (0.096), followed by *Nigella sativa L., Ephedra alata Decne*, *Euphorbia resinifera Berg, Lavandula dentata L.,* and *Berberis hispanica Boiss. & Reut.,* with RFC values of 0.072, 0.054, 0.044, 0.044, and 0.041, respectively (Table 2, Figure 3). The highest RFC values indicated that these species had an increasing traditional interest in the local area for treating cancer. Accordingly, further investigations into these species should be performed to assess phytochemical constituents and pharmaceutical benefits in order to identify their active compounds for any drug formulations [45].

**Figure 4:**
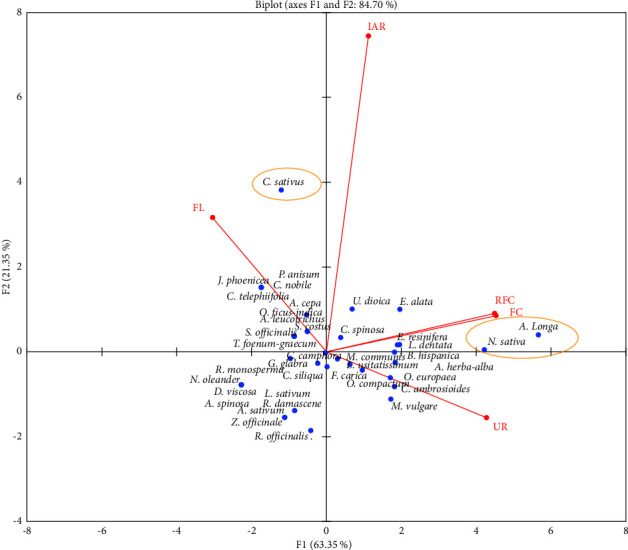
Biplot of principal components analysis for an ethnobotanical index of the reported species.

Principal component analysis (PCA) showed two top principal components (eigenvalue ≥1), accounting for 84.7% of the total variation of parameters studied. To facilitate the selection of species based on the ethnobotanical parameters studied, a biplot was created using two first principal components (F1 and F2). F1 explained 63.35% of the total variation and was strongly influenced by the FC, RFC, UR, and FL. While F2 accounted for 21.35% of the total variation and was significantly influenced by the IAR index, *A. longa* and *N. sativa* were associated positively with RFC, FC, and UR, while *C. sativus* was associated positively with the IAR index (Figure 4). These results indicated the frequent use of *A. longa* and *N. sativa* to treat cancer by the studied population, demonstrating the relative medicinal importance of these two species in the present local area. The common use of *A. longa* and *N. sativa* was already confirmed in Moroccan traditional pharmacopeia, which showed the medicinal values of these two species against cancer as well as related diseases. To that end, efforts must be made to prioritize these species for preservation and protection, as their traditional uses may place the local people under threat due to over-exploitation.

**Figure 5:**
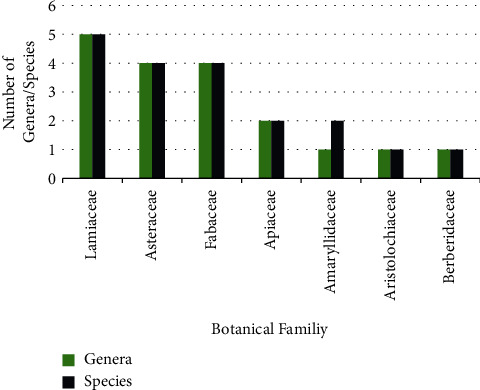
Number of ethnospecies in each family used to treat cancer.

In regard to the plant families, Lamiaceae contributed the highest number of plants with five species (FUV = 0.034), followed by Asteraceae (4 species; FUV = 0.020), Fabaceae (4 species; FUV = 0.020) and Apiaceae (2 species; FUV = 0.13) as shown in Figure 5. This is consistent with our previous studies carried out in different areas in Morocco, including the Rabat-Sale-Kenitra, Fez, and Taounat regions [16, 30, 35], where we found the dominance of the Lamiaceae, Asteraceae, Fabaceae, and Apiaceae families. Moreover, the predominance of these plant families has also been proven in numerous ethnobotanical surveys conducted in other Moroccan regions [38, 45–47], as well as in other neighboring countries like Algeria [48, 49] and Tunisia [50]. Furthermore, the Lamiaceae and Asteraceae families have been specifically reported in traditional medicine against cancer. Besides that, these two botanical families dominate Moroccan flora and are found throughout the country [43].

On the other hand, the Lamiaceae and Asteraceae families have been linked to a variety of pharmacological effects, including antibacterial, antifungal, anti-inflammatory, antidiabetic, antiviral, antioxidant, and insecticidal properties [51–54]. These biological properties are particularly related to their valuable chemical constituents, which have shown promising therapeutic potential [18, 55].

### 3.3. Parts of the Medicinal Plants Used

**Figure 6:**
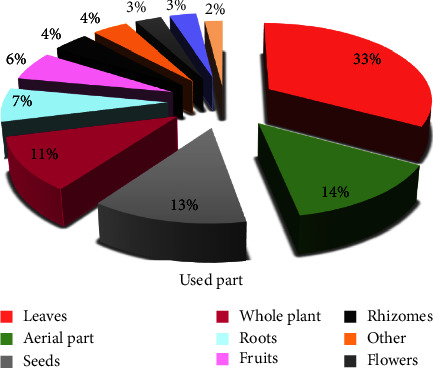
Frequency of different plant parts used.

Different parts of plants are used as medicine to create traditional cancer treatments, including leaves, seeds, stems, fruits, flowers, rhizomes, and roots. The studied population prefers the leaves against cancer (33%), followed by aerial parts (14%), seeds (13%), whole plants (11%), roots (7%), and fruits (6%) (Figure 6). Similarly, ethnobotanical research surveys conducted in Morocco revealed that leaves were a major dominant plant part used in the preparation of traditional remedies [21, 39, 42, 44, 45]. Similar findings were reported in other international ethnobotanical studies in Ghana [56], Lebanon [57], and Turkey [58].

The common traditional use of one part over another varies depending on its active fraction. The availability, ease of harvesting, and simple uses in herbal medicine remedies may explain why leaves were preferred. Furthermore, the leaves are thought to be photosynthesis and secondary metabolite storage sites, which are involved in a variety of health-promoting effects. They contain more phenolic compounds, alkaloids, heterosides, and essential oils. However, when compared to other plant organs such as fruits, roots, and stems, exploiting and harvesting this plant part is a relatively sustainable practice; harvesting the roots may result in the annihilation and disappearance of the plants.

### 3.4. Methods of Remedy Preparations

**Figure 7:**
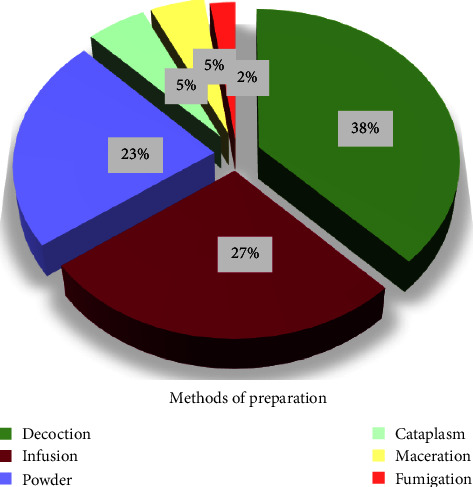
Frequency of different preparation methods.

According to the current ethnobotanical study, several methods are used for remedy preparation.  Figure 7 shows that decoction is the most used method (38%) for herbal preparation against cancer at various stages of development, followed by infusion (27%), and powder (23%). Other methods, such as cataplasm (5%), maceration (5%), and fumigation (2%), are rarely used by the studied population. These findings may be supported by the fact that decoction allows for better extraction of the most active ingredients and reduces or eliminates the toxicity of certain polyherbal prescriptions [38]. The predominance of decoction in our findings is consistent with previous research in the Rabat-Sale-Kenitra, Taounat, and Fez regions [16, 30, 35]. Other ethnobotanical studies in Morocco [38, 42, 44, 45] corroborate these findings.

In terms of administration, the findings revealed that most herbal remedies are taken orally. Other modes of administration, such as inhalation and massage, were also used in some traditional recipes. These findings have also been reported in national and international traditional pharmacopeias, indicating that the oral route is the most common and acceptable mode for patients to receive plants [39, 48, 49, 58].

### 3.5. Cancer Categories and Their FIC Values

Plants identified in this survey were used to treat nine types of cancer, which can be classified as brain, gastrointestinal, kidney, leukemia, oral, pancreatic, prostate, skin, and uterine cancer.

In this survey, plants identified were used to treat nine categories of cancer, which can be grouped into brain, gastrointestinal, kidney, leukemia, oral, pancreatic, prostate, skin, and uterine cancer. The informant consensus factor (FIC) values of the claimed plants in this survey ranged between 0.36 and 0.83 (Table 3), indicating a lack of consensus on patients suffering from a specific type of cancer in comparison to traditional knowledge, as well as a lack of knowledge exchange among people in different areas [59]. This disparity in knowledge among the cancer patients studied may be explained by their belief that all types of cancer are the same disease. In contrast, certain cancer types, such as uterine and skin cancer, showed interesting agreement among patients regarding the use of plant species (FIC values of 0.83 and 0.6, respectively). These highest FIC values demonstrated reasonable reliability in agreement with the use of plants against cancer.

### 3.6. Scientific Evidence and Mechanisms of Action of Anticancer Medicinal Plants

In this study, the 39 identified plants against cancer have been pharmacologically validated through *in vitro* and *in vivo* studies [16, 60–65]. In Table 4, we listed the mechanisms of anticancer properties of the inventoried plants used traditionally against cancer by the studied population.

**Figure 8:**
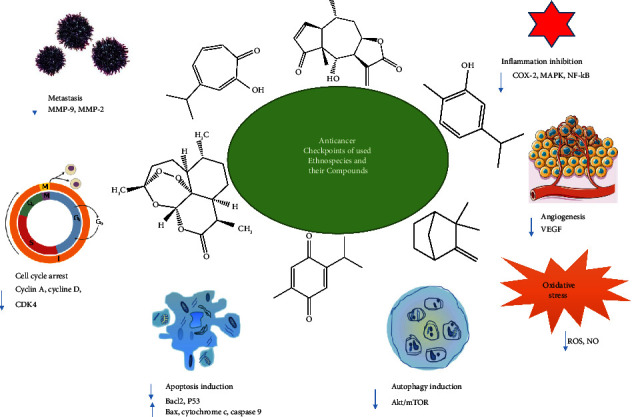
Anticancer molecular targets of reported medicinal plants and their bioactive molecules.

These medicinal plants have shown promising anticancer properties targeting multiple checkpoints, including angiogenesis, inflammation inhibition, cell cycle arrest, metastasis, induction of cell apoptosis, and autophagy, thanks to their valuable chemical constituents (Figure 8). *A. longa* is the most frequently used medicinal plant by informants to treat cancer. Indeed, aqueous extracts of *A. longa* have been shown to induce cell death in a dose-dependent manner, resulting in cell apoptosis associated with a loss of mitochondrial membrane integrity and the up-regulation of caspases-9 and -3 followed by PARP cleavage [48]. Furthermore, *C. sativus* ethanol extracts demonstrated promising anticancer properties in MCF-7 breast cancer cells [84]. These extracts induced caspase-dependent apoptosis by increasing the expression of the Bax protein. Furthermore, n-hexane extracts from *Retama monosperma* leaves induced cell cycle arrest and extrinsic pathway-dependent apoptosis in Jurkat cells [82]. *Trigonella foenum-graecum* seeds inhibited angiogenesis in HepG2 cells, inducing cell cycle arrest at the G2/M phase after 12 and 48 hours of treatment, and a remarkable arrest at the G1/S phase after 24 hours [83]. *Ephedra alata* is another species with anticancer potential, significantly inhibiting cell growth and migration in 4T1 breast cancer cells in a dose and time-dependent manner [78].

This effect was mediated by the induction of apoptosis and caspase activation, as well as the up-regulation of reactive oxygen species (ROS) production [78]. Other Asteraceae plant species, such as *Artemisia herba-alba*, *Chamaemelum nobile*, *Saussurea costus*, and *Dittrichia viscosa*, demonstrated significant anticancer properties against various cell lines, with IC_50_ values ranging from 20.07 to 168.40 g/mL [62, 70–72]. Artemisinin, a major naturally occurring compound found in *A. herba-alba*, is thought to be an effective anticancer agent. Artemisinin's anticancer activity has been linked to several molecular mechanisms, including apoptosis induction, cell cycle arrest, metastasis inhibition, and increased oxidative stress via ROS and NO production [96, 97].

**Figure 9:**
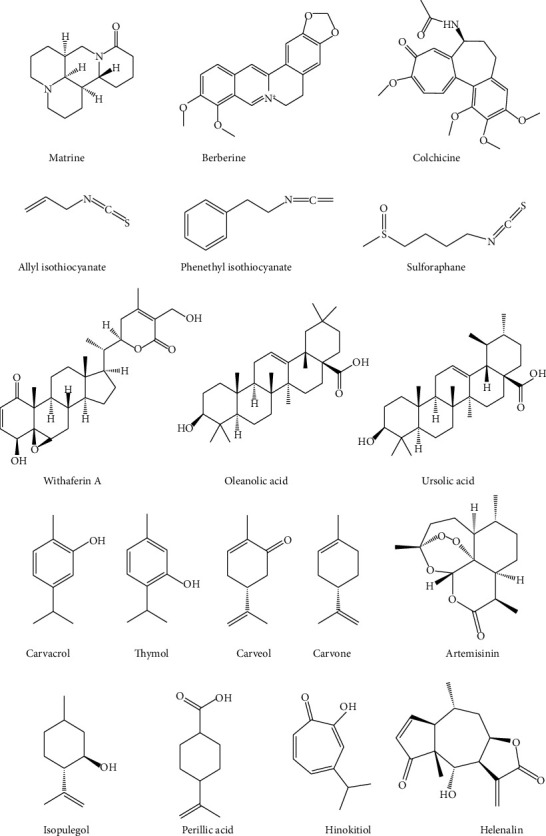
Some phytochemical compounds with potent anticancer properties.

Plants in the Lamiaceae family, on the other hand, are distinguished by the presence of specific structures involved in the production and secretion of volatile compounds as secondary metabolites, such as thymol, carvacrol, carvone, carveol, and hinokitiol (Figure 9). Several plants, including *Lavandula dentata*, *Marrubium vulgare*, *Origanum compactum*, *Rosmarinus officinalis*, and *Salvia officinalis*, contain these compounds, which are responsible for their anticancer properties. Essential oils extracted from the aerial parts of *S. officinalis* reduced cell viability in breast MCF7 cancer, human LNCaP prostate adenocarcinoma, and HeLa cell lines after a 48-hour incubation at concentrations of 100 and 200 g/mL [89]. Furthermore, *O. compactum* essential oils have high cytotoxicity against MCF-7 cells, with an IC_50_ of 113.42 ± 4.6 g/mL [87].

Carvacrol and thymol, remarkably, have been assigned anticancer potential against various cancer cell lines, with varying modes of action [98–100]. Indeed, thymol has been shown to inhibit the G0/G1 phase transition of the cell lines cellosaurus P-815 and MCF-7 [100]. In human promyelocytic leukemia (HL-60) cells, thymol induces both caspase-dependent and caspase-independent apoptosis [98]. Carvacrol has also been identified as an effective anticancer agent, inducing cell apoptosis in HepG2 cell lines via caspase activation and the mitogen-activated protein kinase (MAPK) pathway [101]. Furthermore*, in vivo* studies showed that carvacrol has the potential to prevent liver cancer in male Wistar albino rats induced by diethylnitrosamine [99].

## 4. Conclusions

This ethnobotanical survey revealed that the locals have extensive indigenous knowledge of the use of medicinal plants, particularly for cancer treatment. These valuable traditional practices reflect the rich and varied floristic patrimony of the studied area. More efforts, however, must be made to prioritize certain medicinal plants for preservation and protection, as their preferred traditional uses may endanger them due to over-exploitation. Furthermore, based on the claimed results and suggested discussions, some identified medicinal plants may have a promising role in the treatment of various types of cancer at various stages of development, including prostate, gastrointestinal, uterine, leukemia, brain, skin, oral, kidney, and pancreatic cancer. However, this medicinal value requires further clarification and should be used with caution. Further research into these plant species is needed to assess phytochemical constituents and pharmaceutical benefits in order to identify active compounds for any drug formulations. Furthermore, data on the toxicity of medicinal plants is essential for ensuring the safety of their uses as well as standardizing their accurate posology and therapeutic doses.

## Figures and Tables

**Table 1:** Informant sociodemographic profile.

Sociodemographic characteristic	Variables	Numbers of informants (%)	Number of used ethnospecies (%)	*p* value
Age	20–40	85(29%)	9(53.84%)	0.001 ^*∗*^
	40–60	159(55%)	21(53.84%)	
	More than 60	47(16%)	33(84.61%)	

Gender	Male	128(44%)	26(66.66%)	0.140
	Female	163(54%)	37(94.87%)	

Education level	No	105(36%)	29(74.35%)	0.360
	Primary	67(23%)	32(82.05%)	
	Secondary	81(28%)	17(43.58%)	
	University	38(13%)	8(20.51%)	

Location	Rural	176(60%)	37(94.87%)	0.140
	Urban	125(40%)	30(76.92%)	

Socio-economic situation	High	14(5%)	18(46.15%)	0.424
	Intermediate	148(51%)	27(29.23%)	
	Low	129(44%)	34(87.17%)	

Values with ^*∗*^ indicate the significant correlation between the number of reported species with the age variable ( ^*∗*^*p*  <  0.05).

**Table 2:** Herbal remedies used by local people for the treatment of cancer.

Family	Scientific name (EPPO code)	Voucher	Vernacular name Arabe (English)	Used part	Method of preparation/Route of administration	Common traditional dosages	FL (%)	FC	UR	RFC	FUV	IAR	Toxicity (lethal dose (LD_50_))	Revised literature
Amaryllidaceae	*Allium cepa* L. (ALLCE 23211)	BLMUP306	Bassela (onion)	Bulbs	Inf, Dec/In	Glass	60% brain; 40% leukemia	5	2	0.017	0.012	0.75	LD_50_ = 3000 mg/kg b.w. (body weight)	[35]
	*Allium sativum* L. (ALLSA 23233)	BLMUP307	Toma (garlic)	Bulbs	Dec, Mac/In	3 Units	50% oral; 50% pancreatic	2	2	0.006		ND	LD_50_ = 5000 mg/kg b.w	[16]

Apiaceae	*Ammodaucus leucotrichus* coss. (AMKLE 23511)	BLMUP308	Kamoun essoufi (wooly cumin)	Fruits	Pow/In	Spoon	75% oral; 25% prostate	4	2	0.013	0.009	0.66	LD_50_ = 520 mg/kg b.w	[35]
	*Pimpinella anisum* L. (PIMAN40505)	BLMUP309	Habat hlawa (anise)	Seeds	Dec, Pow/In	Spoon, pinch	100% gastro-intestinal	2	1	0.006		1	LD_50_ = 25 ± 2 g/kg b.w	[39]

Apocynaceae	*Nerium oleander* L. (NEROL 38433)	BLMUP310	Dafla (oleander)	Leaves, flowers	Dec/Ex	Handful	100% skin cancer	1	1	0.003	0.003	ND	LD_50_ = 57 mg/Kg b.w	[16]

Aristolochiaceae	*Aristolochia longa* L. (ARPLO 24116)	BLMUP311	Berztem (pipevine)	Roots, whole plant	Dec, Pow/In, Ex	Handful spoon, pinch	35% uterine cancer; 21% skin cancer	28	7	0.096	0.10	0.77	LD_50_ = 407.38 mg/kg b.w	[40]

Asteraceae	*Artemisia herba-alba* Asso (ARTHA 24166)	BLMUP312	Chih (white mugwort)	Aerial parts, leaves	Inf, Dec/In, Ex	Coffee spoon	36% pancreatic	11	5	0.037	0.014	0.66	LD_50_ = 2600 mg/kg b.w	[30]
	*Chamaemelum nobile* (L.) All. (ANTNO 23736)	BLMUP313	Babonj (chamomile)	Flowers	Inf/In	Spoon	100% gastro-intestinal	2	1	0.006		1	LD_50_ = 5000 mg/kg b.w	[41]
	*Dittrichia viscosa* (L.) Greuter (INUVI 34981)	BLMUP314	Magraman (false yellowhead)	Aerial parts	Dec/In	ND	100% brain cancer	1	1	0.003		ND	LD_50_ = 1720.25 mg/kg/b.w.	[41]
	*Saussurea costus* (Falc.) Lipsch. (SAULA 44090)	BLMUP315	Quist AL-Hindi (Indian costus)	Whole plant	Pow/In	Pinch	75% uterine cancer	4	2	0.013		0.66	LD_50_ > 1000 mg/kg b.w	[16]

Berberidaceae	*Berberis hispanica* Boiss. & Reut. (BEBHI 25054)	BLMUP316	Aghris (barberry)	Roots	Pow, Dec/In, Ex	Glass	33% uterine; 33% skin	12	4	0.041	0.041	0.72	LD_50_ = 1016.16 mg/kg b.w	[40]

Brassicaceae	*Lepidium sativum* L. (LEPSA 35947)	BLMUP317	Hab-errachad (garden cress)	Seeds, leaves	Pow, Mac/In	Pinch	100% prostate	1	1	0.003	0.003	ND	LD_50_ = 639 mg/kg b.w	[41]

Cactaceae	*Opuntia ficus-indica* (L.) Mill. (OPUFI 39060)	BLMUP318	Hindia (barbary fig)	Fruits, whole plants	Dec, Inf/In, Ex	Glass	66% gastro-intestinal	5	2	0.017	0.017	0.75	LD_50_ = 2.72 ml/kg body	[21]
Capparaceae	*Capparis spinosa* L. (CPPSP 28130)	BLMUP319	Kabar (flinders rose)	Leaves, fruits	Pow/Ex	Spoon	62% gastro-intestinal; 25% pancreatic	8	3	0.027	0.027	0.71	LD_50_ = 5140 mg/kg b.w	[42]

Caryophyllaceae	*Corrigiola telephiifolia* Pourr. (CGLTE 26812)	BLMUP320	Serghina (corrigiola)	Whole plant, leaves	Dec/In	Coffee spoon	83% uterine; 17% kidney	6	2	0.020	0.020	0.8	LD_50_ = 14.000 mg/kg b.w	[42]

Chenopodiaceae	*Chenopodium ambrosioides* L. (CHEAM 26847)	BLMUP321	Mkhinza (sweet pigweed)	Leaves	Dec/In, Ex	Spoon	40% uterine; 20% skin; 10% prostate; 10% pancreatic	10	6	0.034	0.034	0.44	LD_50_ = 460 mg/kg b.w	[43]

Cupressaceae	*Juniperus phoenicea* L. (IUPPH 35388)	BLMUP322	Aaraar (phoenician juniper)	Aerial parts	Pow, Dec/In, Ex	Spoon	100% kidney	2	1	0.006	0.006	1	LD_50_ = 3432 mg/kg b.w	[16]

Ephedraceae	*Ephedra alata* Decne (EPEAL 86676)	BLMUP323	Andla (ephedra)	Leaves	Inf, Pow/In, Ex	Spoon	ND	16	5	0.054	0.054	0.73	LD_50_ = 500 mg/kg b.w	[42]

Euphorbiaceae	*Euphorbia resinifera* O. Berg (EPHRN 31428)	BLMUP324	Daghmous (spurge)	Leaves	Pow, Dec/In, Ex	Pinch	38% skin; 38% uterine; 15% gastro-intestinal;	13	4	0.044	0.044	0.75	LD_50_ > 2.5 mg/kg b.w	[42]
Fabaceae	*Ceratonia siliqua* L. (CEQSI 26680)	BLMUP325	SAnnâ hram (carob)	Leaves	Dec/In	Glass	60% skin; 20% oral; 20% gastro-intestinal	5	3	0.017	0.011	0.5	LD_50_ > 5000 mg/kg b.w	[44]
	*Glycyrrhiza glabra* L. (GYCGL 33535)	BLMUP326	Araq-sűs (liquorice)	Roots, stems	Pow, Dec/In	Spoon	66% leukemia; 33% skin	3	2	0.010		0.5	DL50 > 1000 mg/kg b.w	[21]
	*Retama monosperma* (L.) Boiss. (RTARE98145)	BLMUP327	R'tum (bridal broom)	Aerial parts	Dec/In	Pinch	100% oral cancer	1	1	0.003		ND	LD_50_ = 1995 mg/kg b.w	[41]
	*Trigonella foenum-graecum* L. (TRKFG47367)	BLMUP328	Helba (fenugreek)	Seeds	Pow/In	Pinch	75% oral; 25% gastro-intestinal	4	2	0,013		0.66	LD_50_ = 7000 mg/kg b.w	[39]

Iridaceae	*Crocus sativus* L. (CVOSA28993)	BLMUP329	Safran (saffron)	Roots	Pow/In, Ex	Spoon	100% leukemia	3	1	0.010	0.010	2	LD_50_ = 1.48 ml/kg b.w	[39]

Lamiaceae	*Lavandula dentata* L. (LAVDE35748)	BLMUP330	Khzama (fringed lavender)	Leaves	Dec/In	Spoon	ND	13	7	0.044	0.024	0.5	LD_50_ = 2000 mg/kg b.w	[23]
	*Marrubium vulgare* L. (MAQVU 37199)	BLMUP331	Marwita (white horehound)	Leaves, Aerial parts	Dec/In, Ex	Glass	33% uterine; 22% oral	9	6	0.030		0.37	LD_50_ > 2000 mg/kg b.w	[39]
	*Origanum compactum* Benth. (ORICO93447)	BLMUP332	Zaatar (oregano)	Aerial parts	Dec, Inf/In	Handful	37% gastro-intestinal; 37% oral	8	4	0.027		0.57	LD_50_ > 5000 mg/kg b.w	[16]
	*Rosmarinus officinalis* L. (RMSOF 43318)	BLMUP333	Azir (rosemary)	Aerial parts	Dec, Inf/In	Glass	33% oral; 33% pancreatic; 33% prostate	3	3	0.010		ND	LD_50_ = 561 mg/kg b.w	[35]
	*Salvia officinalis* L. (SALOF44001)	BLMUP334	Salmiya (sage)	Leaves	Inf, Dec/In	Glass	75% pancreatic; 25% oral	4	2	0.013		0.66	LD_50_ = 1287.3 mg/Kg b.w	[35]

Linaceae	*Linum usitatissimum* L. (LIUUT 36286)	BLMUP335	Zeri-it-lktan (flax seed)	Seeds	Pow/In, Ex	Coffee spoon	28% oral; 28% uterine; 14% leukemia	7	3	0.024	0.024	0.66	LD_50_ = 3.76 mg/Kg b.w	[23]

Lauraceae	*Cinnamomum camphora* (L.) J. Presl (CINCA 27139)	BLMUP336	Kafour (camphor)	Whole plant	Fum/Ex	Spoon	30% brain; 20% skin	5	2	0.017	0.017	0.75	LD_50_ = 5100 mg/kg b.w	[23]

Moraceae	*Ficus carica* L. (FIUCA 32494)	BLMUP337	Karmous (fig)	Fruits	Pow/In	Spoon	75% uterine; 12% skin; 12% pancreatic;	6	4	0.020	0.020	0.4	LD_50_ > 6000 mg/kg b.w	[21]

Myrtaceae	*Myrtus communis* L. (MYVCO 38308)	BLMUP338	Rihan (true myrtle)	Leaves	Inf, Fum/In, Ex	Handful	33% pancreatic; 33% skin	8	3	0.027	0.027	0.71	LD_50_ = 473 mg/kg b.w	[42]

Oleaceae	*Olea europaea* L. (OLVEU38940)	BLMUP339	Zitoun (olive)	Leaves	Dec, Inf/In, Ex	Glass	30% pancreatic; 20% kidney	10	5	0.034	0.034	0.55	LD_50_ = 3475 mg/kg b.w	[35]

Renonculaceae	*Nigella sativa* L. (NIGSA 38473)	BLMUP34	Sanouj (black cumin)	Seeds	Pow, Dec/In, Ex	Coffee spoon	24% skin; 14% gastro-intestinal	21	6	0.072	0.072	0.75	LD_50_ = 1,853 mg/kg b.w	[40]

Rosaceae	*Rosa* × *damascene* Herrm. (ROSDM 43433)	BLMUP341	Ward-lbeldi (damask rose)	Flowers	Dec, Fum/Ex	Handful	66% skin; 33% gastro-intestinal	3	3	0.010	0.010	ND	LD_50_ > 2500 mg/kg b.w	[16]

Sapotaceae	*Argania spinosa* (L.) Skeels (ARJSI 24031)	BLMUP342	Argane (argan)	Fruits	Pow, Cat/In, Ex	Glass	100% pancreatic	1	1	0.003	0.003	ND	LD_50_ = 1300 mg/kg b.w	[44]

Urticaceae	*Urtica dioica* L. (URTDI 47803)	BLMUP343	Harriga (common nettle)	Aerial parts	Dec, Cat/In, Ex	Handful	64% uterine; 36% skin	11	2	0.037	0.037	0.9	LD_50_ = 3625 mg/kg b.w	[44]

Zingiberaceae	*Zingiber officinale* Roscoe (ZINOF 48967)	BLMUP344	Skinjbir (ginger)	Rhizomes	Pow/In	Glass	50% kidney; 50% gastro-intestinal	2	2	0.006	0.006	ND	LD_50_ = 4525.5 mg/kg b.w	[16]

Inf, infusion; Dec, decoction; Pow, powder; Mac, maceration; Fum, fumigation; Cat, cataplasm; In, internal uses, Ex, external uses.

**Table 3:** Cancer categories and their FIC values.

Cancer type	Nur	Nt	F_IC_	Plants species (FC)
Brain cancer	9	5	0.5	*Dittrichia viscosa* (L.) Greuter **(1)**, *Allium cepa* L. **(3)**, *Artemisia herba-alba* Asso. **(1)**, *Cinnamomum camphora* (L.) J. Presl, **(3)**, *Origanum compactum* Benth. **(1)**

Gastro-intestinal cancer	34	16	0.54	*Aristolochia longa* L. **(3)**, *Zingiber officinale* Roscoe **(1)**, *Ficus carica* L. **(1)**, *Pimpinella anisum* L. **(2)**, *Chamaemelum nobile* (L.) All. **(2)**, *Capparis spinosa* L. **(5)**, *Saussurea costus* (Falc.) Lipsch. **(1)**, *Opuntia ficus-indica* (L.) Mill. **(4)**, *Chenopodium ambrosioides* L. **(1)**, *Trigonella foenum-graecum* L. **(1)**, *Rosa* *×* *damascene* Herrm, **(1)**, *Nigella sativa* L. **(3)**, *Marrubium vulgare* L. **(1)**, *Origanum compactum Benth*. **(3)**, *Ceratonia siliqua* L. **(1)**, *Euphorbia resinifera* O. Berg. **(2)**

Kidney cancer	14	8	0.46	*Aristolochia longa* L. **(1)**, *Ficus carica* L **(1)**, *Corrigiola telephiifolia* Pourr. **(1)**, *Juniperus phoenicea* L. **(2)**, *Zingiber officinale* Roscoe (**1)**, *Artemisia herba-alba* Asso **(2)**, *Berberis hispanica* Boiss. & Reut. **(3)**, *Olea europaea* L. **(2)**

Leukemia	12	7	0.45	*Linum usitatissimum* L. **(1)**, *Allium cepa* L. **(2)**, *Crocus sativus* L. **(3)**, *Glycyrrhiza glabra* L. **(2)**, *Nigella sativa* L. **(1)**, *Euphorbia resinifera* O. Berg, **(1)**, *Aristolochia longa* L. **(2)**

Oral cancer	27	14	0.5	*Olea europaea* L. **(4)**, *Linum usitatissimum* L. **(2)**, *Salvia officinalis* L. **(1)**, *Allium sativum* L. **(1)**, *Ammodaucus leucotrichus* Coss. **(3)**, *Ceratonia siliqua* L. **(1)**, *Origanum compactum* Benth. **(3)**, *Retama monosperma* (L.) Boiss. **(1)**, *Marrubium vulgare* L. **(2)**, *Trigonella foenum-graecum* L. **(3)**, *Artemisia herba-alba* Asso, **(1)**, *Chenopodium ambrosioides* L. **(1) ***Rosmarinus officinalis* L. **(1) ***Nigella sativa* L. **(2)**

Pancreatic cancer	24	14	0.43	*Argania spinosa* (L.) Skeels **(1)**, *Aristolochia longa* L. **(1)**, *Capparis spinosa* L. **(2)**, *Chenopodium ambrosioides* L. **(1)**, *Rosmarinus officinalis* L. **(1)**, *Nigella sativa* L. **(1)**, *Ficus carica* L. **(2)**, *Salvia officinalis* L. **(3)**, *Allium sativum* L. **(1)**, *Artemisia herba-alba* Asso. **(4)**, *Myrtus communis* L. **(1)**, *Olea europaea* L. **(3)**, *Marrubium vulgare* L. **(1)**, *Origanum compactum* Benth. **(2)**

Prostate cancer	12	8	0.36	*Aristolochia longa* L. **(5)**, *Rosmarinus officinalis* L. **(1)**, *Lepidium sativum* L. **(1)**, *Ammodaucus leucotrichus Coss*. **(1)**, *Berberis hispanica* Boiss. & Reut. **(1)**, *Chenopodium ambrosioides* L. **(1)**, *Olea europaea* L. **(1)**, *Marrubium vulgare* L. **(1)**

Skin cancer	44	18	0.6	*Aristolochia longa* L. **(6)**, *Glycyrrhiza glabra* L. **(1)**, *Nigella sativa* L. **(5)**, *Olea europaea* L. **(1)**, *Myrtus communis* L. **(1)**, *Urtica dioica* L. **(4)**, *Chenopodium ambrosioides* L. **(2)**, *Nerium oleander* L. **(1)**, *Ficus carica* L. **(2**), *Marrubium vulgare* L. **(1)**, *Euphorbia resinifera* O. Berg, **(5)**, *Cinnamomum camphora* (L.) J. Presl, **(2)**, *Rosa × damascene* Herrm. **(1)**, *Artemisia herba-alba* Asso. **(2)**, *Opuntia ficus-indica* (L.) Mill. **(2)**, *Berberis hispanica* Boiss. & Reut. **(4)**, *Capparis spinosa* L. **(1)**, *Ceratonia siliqua* L. **(3)**

Uterine cancer	60	11	0.83	*Aristolochia longa* L. **(10)**, *Nigella sativa* L. **(11)**, *Myrtus communis* L. **(6)**, *Urtica dioica* L. **(7)**, *Euphorbia resinifera* O. Berg, **(5)**, *Linum usitatissimum* L. **(2) ***, Saussurea costus* (Falc.) Lipsch. **(3)**, *Corrigiola telephiifolia* Pourr. **(5)**, *Chenopodium ambrosioides* L. **(4)**, *Marrubium vulgare* L. **(3)**, *Berberis hispanica* Boiss. & Reut. **(4)**

Nur, number of use report; Nt, number of plant species used by category.

**Table 4:** Anticancer properties of the medicinal plants cited in the study area.

Plant species	Used parts	Used extracts	Experimental model	Key results	References
*Allium cepa* L.	Bulbes	Ethanol, methanol, aqueous extracts	HepG2 human liver cancer cell lines HeLa cervical cancer cell line	Exhibited significant antigenotoxic activity in HepG2 cells decreased the levels of intracellular ROS at 1–100 *μ*g/mL concentrations. Showed anticancer effect on HeLa cells (IC_50_ = 4.8 *μ*M)	[66, 67]

*Allium sativum* L.	Bulbes	Ethanol	Breast (MDAMB231), prostate (PC3), colon (HCT-15), hepatic cancer (Hep3B) cell lines	IC_50_ (MDAMB231) = 5.748 mg/mL IC_50_(PC3) = 3.333 mg/mL IC_50_(HCT-15) = 3.746 mg/mL IC_50_(Hep3B) = 3.301 mg/mL	[65]

*Ammodaucus leucotrichus* coss.	Aerial parts	Essential oil	HCT116 colon cancer cell line HePG2 (human hepatocellular carcinoma cell line	IC_50_ = 72.6 *μ*g/mL and 110.6 *μ*g/mL, respectively	[64]

*Pimpinella anisum* L.	Seeds	Alcoholic extracts and essential oil	Gastric cancer cell line	IC_50_ = 30 *µ*g/mL	[68]

*Nerium oleander* L.	Flowers	Essential oil	Ehrlich ascites carcinoma cells (EAC)	Inhibited significantly the growth of EAC cells	[60]

*Aristolochia longa* L.	Roots	Aqueous extract	Burkitt's lymphoma BL41 cells	Induced cell death in a dose-dependent manner triggered cell apoptosis, associated with a loss of mitochondrial membrane integrity and the upregulation of caspases-9 and -3 followed by PARP cleavage	[69]

*Artemisia herba-alba* Asso	Aerial parts	Essential oil	P815 murin mastocytoma and BSR kidney adenocarcinoma of hamster cell lines	Exhibited significant cytotoxic effect	[70]

*Chamaemelum nobile* (L.) All.	Aerial part	Aqueous extract	MCF7	IC_50_ = 168.40 *μ*g/mL	[71]

*Dittrichia viscosa* (L.) Greuter	Aerial part	Ethanol extract	Human breast cancer (estrogen receptor-negative; MDA-MB-231) and prostate cancer (PC3) cell lines	Significantly suppressed cell proliferation and induced cell apoptosis in all the studied cell lines	[72]

*Saussurea costus* (Falc.) Lipsch.	Roots	Hexane extract	Human colon (HCT116) cells	IC_50_ of 20.07 ± 7.25 *μ*g/mL	[62]

*Berberis hispanica* Boiss. & Reut	Roots	Methanol extracts	Breast MDA-MB-231 and MCF-7 cell lines	Exhibited significant antiproliferative potential with IC_50_ values of 6.55 ± 0.58 and 17.95 ± 0.58 *µ*g/mL respectively	[63]

*Lepidium sativum* L.	Leaves	Aqueous extract	Cellosaurus CAL-27 cell lines	Induced noticeable damage to DNA, and up-regulated the levels of mitochondrial reactive oxygen species (ROS), leading to apoptosis (up to 30% and 60%)	[73]

*Opuntia ficus-indica* (L.) Mill.	Fruit	Ethanol extract	PC3 and MCF7	IC_50_ values of 5775.7 and 6311.3 *μ*g/ml, respectively	[74]

*Capparis spinosa* L.	Leaves	Ethanolic extract	Ehrlich ascites carcinoma in Swiss albino mice	Induced apoptosis through the activation of caspase 3 and BCL2 proteins	[75]

*Corrigiola telephiifolia* Pourr	Roots	Dichloromethane extract	Murine colon adenocarcinoma (CT-26) cells	IC_50_ = 80 ± 4.56	[61]

*Chenopodium ambrosioides* L.	Leaves	EO	Liver cancer SMMC-7721 cells	Inhibited cell proliferation, and cell cycle division at Go/G1 phase. Caused caspase-dependent apoptosis	[76]

*Juniperus phoenicea* L.	Aerial parts	Chloroform fraction	MCF-7 cancer cell	Suppressed cell proliferation, induced cell cycle arrest at the G1 phase, and cell apoptosis	[77]

*Ephedra alata* Decne	Aerial parts	Methanol extract	4T1 breast cancer cells	Inhibited cell growth and migration in a dose and time-dependent manner and triggered apoptosis, reduced ROS production, and induced caspases activation	[78]

*Euphorbia resinifera* O. Berg	Aerial parts	Hexane extract	Rhabdomyosarcoma cancerous, kidney adenocarcinoma of hamster and monkey kidney cancerous cell lines	IC_50_ values between 50.7 ± 4,89 and 266.43 ± 10.20 *µ*g/mL	[79]

*Ceratonia siliqua* L.	Seeds	Ethanol extract	Human glioblastoma cancer cells	Decreased cell viability in a concentration-dependent manner	[80]

*Glycyrrhiza glabra* L.	Roots	Methanol extract	Carcinoma (Caco-2) and prostate carcinoma (PC-3) cell lines	IC_50_ values ranged between 40 and 40.6 *μ*g/ml	[81]

*Retama monosperma* (L.) Boiss.	Leaves	n-Hexane extract	Jurkat cells	Promoted cell cycle arrest and triggered extrinsic pathway-dependent apoptosis	[82]

*Trigonella foenum-graecum* L.	Seeds	Ethanol extract	HepG2 cells	Showed antiangiogenic activity, inducing cell cycle arrest at the G2/M phase after 12 and 48 h of treatment and remarkable arrest at the G1/S phase after 24 h of treatment	[83]

*Crocus sativus* L.	Flowers	Ethanol extract	Breast cancer cells MCF-7	Caspase activation and increased expression of Bax protein lead to cell apoptosis	[84]

*Lavandula dentata* L.	Aerial parts	Dichloromethane extract	Erlish cell line	Showed interesting antitumor activity at a concentration of 300 *µ*l/ml	[85]

*Marrubium vulgare* L.	Aerial parts	Methanol extract	Jurkat cells	IC_50_ > 50 *μ*g/mL	[86]

*Origanum compactum* Benth.	Aerial parts	Ethyl acetate extract	MCF-7 cells	IC_50_ = 279.51 ± 16 *µ*g/mL	[87]

*Rosmarinus officinalis* L.	Aerial parts	EO	Rhabdomyosarcoma cells	IC_50_ = 113.42 ± 4.6 *µ*g/mL	[88]

*Salvia officinalis* L.	Aerial parts	EO	MCF7, LNCaP, and HeLa cells	Reduced cell viability after a 48-hour incubation at concentrations of 100 and 200 *μ*g/mL	[89]

*Linum usitatissimum* L.	Seeds	Methanol extract and ethyl acetate fraction	HepG2 and MCF7 cells	IC_50_ = 60 ± 0.24 *μ*g/ml (HepG2) IC_50_ = 29.4 ± 0.12 *μ*g/ml (MCF7) IC_50_ = 94.7 ± 0.21 *μ*g/ml IC_50_ = 227 ± 0.48 *μ*g/ml, respectively	[90]

*Cinnamomum camphora* (L.) J. Presl	ND	ND	Cervical carcinoma HeLa cells	Pinoresinol inhibited significantly cell growth and proliferation	[91]

*Ficus carica* L.	Latex	ND	MDA-MB-231 cells	Exhibited promising antimetastatic effects through ERK2, CREB, and AKT2 signaling pathways. Showed genotoxic and cytotoxic effects in MDA-MB-231 cells	[92]

*Argania spinosa* (L.) Skeels	Fruits	Methanol n-butanol extracts	HTC hepatoma cell lines	Significant antiproliferative effect inhibition of ERK1/2 activation was also associated with decreased DNA synthesis	[93]

*Urtica dioica* L.	Roots	Methanolic extract	Balb/c mouse model of benign prostatic hyperplasia	Oral treatment at a dose of 5 mg for 28 days reduced hyperplasia with 51.4% growth inhibition	[94]

*Zingiber officinale* Roscoe	Roots	EO	Swiss albino mice	Significantly reduced the volume of solid tumor development by 54.4% At a concentration of 500 mg/kg body weight	[95]

EO, essential oil.

## Data Availability

All the data supporting the findings of this study are included in this article.
